# Critical transitions in degree mixed networks: A discovery of forbidden tipping regions in networked spin systems

**DOI:** 10.1371/journal.pone.0277347

**Published:** 2022-11-18

**Authors:** Daniel Reisinger, Raven Adam, Marie Lisa Kogler, Manfred Füllsack, Georg Jäger

**Affiliations:** Institute of Systems Sciences, Innovation and Sustainability Research, University of Graz, Graz, Styria, Austria; University of Ottawa Faculty of Engineering, CANADA

## Abstract

Critical transitions can be conceptualized as abrupt shifts in the state of a system typically induced by changes in the system’s critical parameter. They have been observed in a variety of systems across many scientific disciplines including physics, ecology, and social science. Because critical transitions are important to such a diverse set of systems it is crucial to understand what parts of a system drive and shape the transition. The underlying network structure plays an important role in this regard. In this paper, we investigate how changes in a network’s degree sequence impact the resilience of a networked system. We find that critical transitions in degree mixed networks occur in general sooner than in their degree homogeneous counterparts of equal average degree. This relationship can be expressed with parabolic curves that describe how the tipping point changes when the nodes of an initially homogeneous degree network composed only of nodes with degree *k*_1_ are replaced by nodes of a different degree *k*_2_. These curves mark clear tipping boundaries for a given degree mixed network and thus allow the identification of possible tipping intersections and forbidden tipping regions when comparing networks with different degree sequences.

## Introduction

Critical transitions can be conceptualized as abrupt shifts in the state of a system [1]. They typically involve a large change in a system state in response to a small change in an external or critical parameter. Critical transitions are observed in systems from many scientific disciplines including physics [2], ecology [3–7], medicine [8], economics [9], and social science [10] among others [11]. A prominent example from physics is the ferromagnetic transition where a ferrous material shifts from an antiferromagnetic to a ferromagnetic state by spins aligning such that their magnetic moments point in the same direction [2]. An illustrative example from ecology is tipping in shallow lakes where the system abruptly changes from a clear to a turbid state due to changes in nutrient load [4]. Tipping in lakes is also often accompanied by a phenomenon called hysteresis which describes a system’s dependency on its history, making it difficult to restore a system to its original state once it has shifted [12].

Because critical transitions play an important role in such a diverse set of systems it is crucial to understand what parts of a system drive and shape the transition. Theoretic understanding from the field of bifurcation theory shows that critical transition can be induced by qualitative changes in the stability landscape of a system, i.e. the appearance or disappearance of equilibrium points, which in turn is caused by changes in a system’s critical parameter. Many systems prone to such transitions are well understood in terms of their critical parameters and types of bifurcation. For example, the ferromagnetic transition yields a pitchfork bifurcation with respect to changes in temperature and a bifurcation showing the phenomenon of hysteresis with respect to changes in an external magnetic field [2, 10]. The latter is also observed in the shallow lake system with respect to changes in the system’s nutrient load [4].

In addition to identifying critical parameters, a thorough understanding of critical transitions requires an analysis of the underlying network structure of a system, i.e. the way in which the system components are interlinked with each other. Continuing with the example of the ferromagnetic transition, spins are typically assumed to be connected uniformly and regularly on a 2D grid. Changing the temperature across a critical threshold causes the system to shift its state from antiferromagnetic to ferromagnetic. The exact value of this critical threshold depends, besides other system properties, on the network structure of the system. Changing the structure may therefore alter the system’s response to changes in the critical parameter. What is more, many networked systems cannot realistically be assumed to have such simple grid like network structures. Components in these systems may instead be more irregularly connected forming inhomogeneous network structures with varying degrees [13].

In this paper, we investigate how the specifics of the underlying network structure impact the resilience of a system with respect to its critical transition. In this regard, we focus on the effects of changes in the degree sequence of a network, where the degree sequence is simply the collection of node degrees of a network. Varying this property gives us great flexibility in creating a diverse set of networked systems. At the same time, however, it makes it difficult to analyze the systems analytically, for example, through the use of mean field approximations [14]. We therefore employ computer simulations and aggregate our results using Monte Carlo experiments.

The remainder of this paper is organized as follows: First, we introduce a method for generating random networks from a given degree sequence also known as the configuration model [15]. Second, we describe the process of creating critical transitions in arbitrary networked systems using a networked version of the 2D Ising model [16]. Third, we present our results showing how the degree mix in a network’s degree sequence impacts the transition behavior of the networked system. And lastly, we discuss our results and their relevance for evaluating the onset of networked system’s critical transition.

## Methodology

### Configuration model

**Fig 1:**
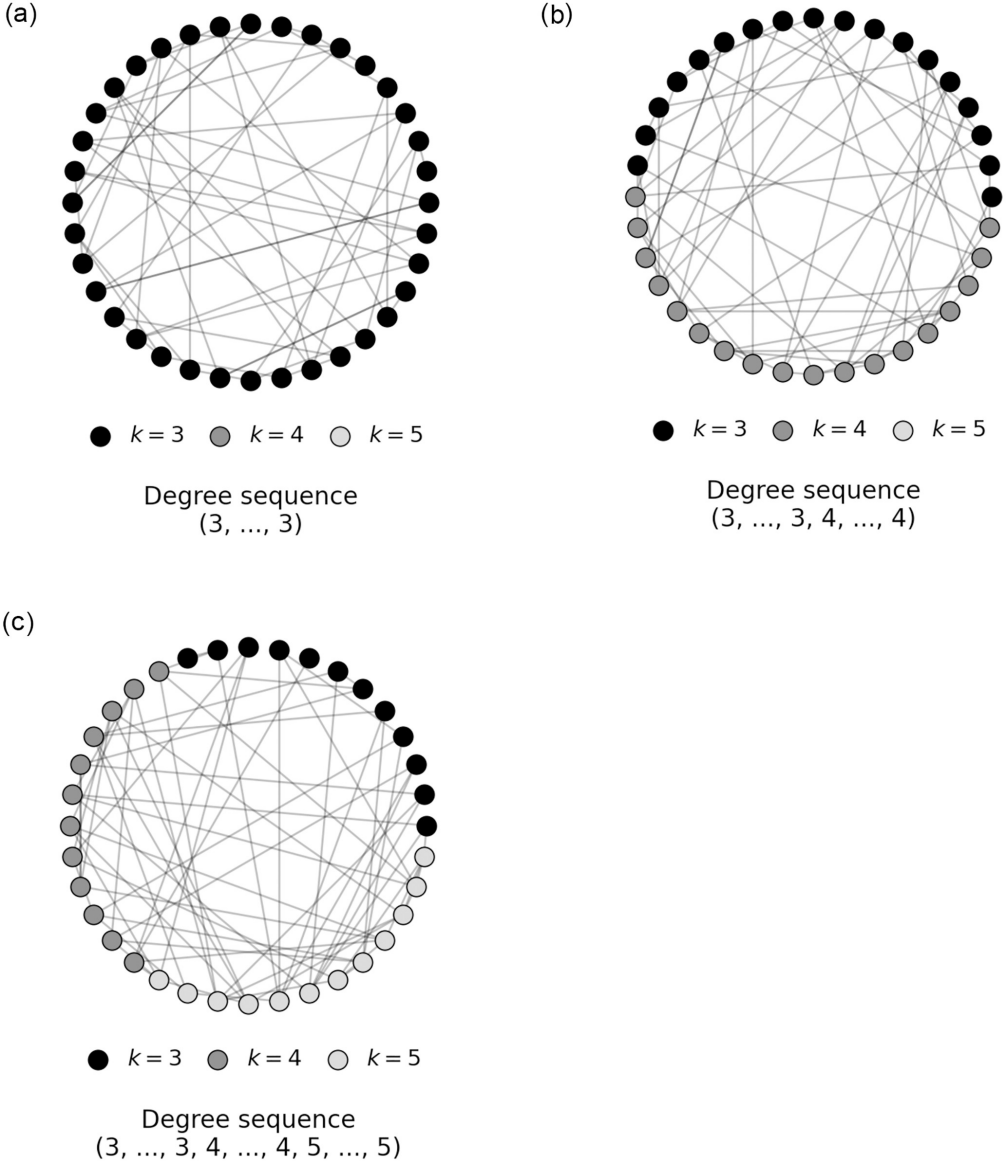
Configuration model. Random networks for different degree sequences constructed using the configuration model. Figure on the left shows configured random network from a degree sequence containing only degree 3. Figure in the middle shows a configured random network from a degree sequence containing degrees 3 and 4. Figure on the right shows a configured random network from degree sequence containing degrees 3, 4 and 5.

Analyzing how mixing of different node degrees effects the shape and onset of a system’s critical transition requires a flexible approach to generate networks. For this purpose, we use the configuration model which allows us to construct random networks from a given degree sequence [15]. Our algorithmic implementation works as follows: We initialize an arbitrary degree sequence (*k*_1_, *k*_2_, …, *k*_*N*_) of length *N* containing for each entry or node an assignment of its degree *k*. To construct a network, the degree sequence must be graphical, i.e. the sum over the degree sequence must be even. If this condition is not satisfied, we randomly remove one of the uneven degrees and proceed by mapping the degree sequence to a list of stubs where a stub is half of a link between two nodes. The list of stubs is then configured into a random network by randomly selecting and connecting two stubs until every stub is paired with another stub. Note that the random network generated in this way may contain self loops, i.e. nodes that are connected to themselves, and multi links, i.e. two nodes that are connected multiple times via different edges. The number of self loops and multi links goes to zero with increasing network length *N* [17]. Some example networks generated by this procedure are shown in Fig 1.

In the context of critical transitions, the degree of a node plays an important role. As experiments showed, it impacts the coupling strength of a networked system and consequently its transition behavior [18]. To capture the changes in coupling strength in response to changes in the degree distribution, we investigate networks generated by a controlled substitution of degrees in its degree sequence. For example, a degree sequence of length *N* initially consisting exclusively of nodes with degree 3 is iteratively substituted by nodes of degree 4 until the whole sequence consists of nodes with degree 4. For each of the possible sequences, we may then construct a random network and investigate changes in the system’s transition behavior.

$$\begin{array}{c} \left(3,\ldots ,3,3,3\right)\\ \left(3,\ldots ,3,3,4\right)\\ \left(3,\ldots ,3,4,4\right)\\ \ldots \\ \left(4,\ldots ,4,4,4\right) \end{array}$$ (1)

Substitution between more than two degree types makes the problem of finding all possible degree sequence combinations computationally more demanding. The example below shows how a degree sequence initially consisting exclusively of nodes with degree 3 is iteratively substituted by nodes of degree 4 and 5 until the whole sequence consists of nodes with degree 5.

$$\begin{array}{c} \left(3,\ldots ,3,3,3\right)\\ \left(3,\ldots ,3,3,4\right)\\ \left(3,\ldots ,3,3,5\right)\\ \left(3,\ldots ,3,4,4\right)\\ \left(3,\ldots ,3,4,5\right)\\ \left(3,\ldots ,3,5,5\right)\\ \ldots \\ \left(5,\ldots ,5,5,5\right) \end{array}$$ (2)

The number of possible combinations is given by the binomial coefficient with *N* being the length of degree sequence or number of nodes and *m* being the number of different degree types.

(N+m-1m-1) (3)

### Ising model

To generate critical transitions, we use a well investigated spin model from statistical physics known as the Ising model [16]. It is exemplary for systems in which coupling mechanisms are at work and it is well understood in terms of its bifurcations [2]. Conceptually, the Ising model has been applied to many contexts outside of its original interest, i.e. the study of ferromagnetism [2]. To name a few examples, in social science and economics the Ising model has been used to study collective phenomena such as opinion dynamics and financial crisis [10, 19]. In ecology, it has been used to explain spatial patterns in tree yield [20]. And in biology, it has been used to study the brain, cancer, and protein folding [21–24].

**Fig 2:**
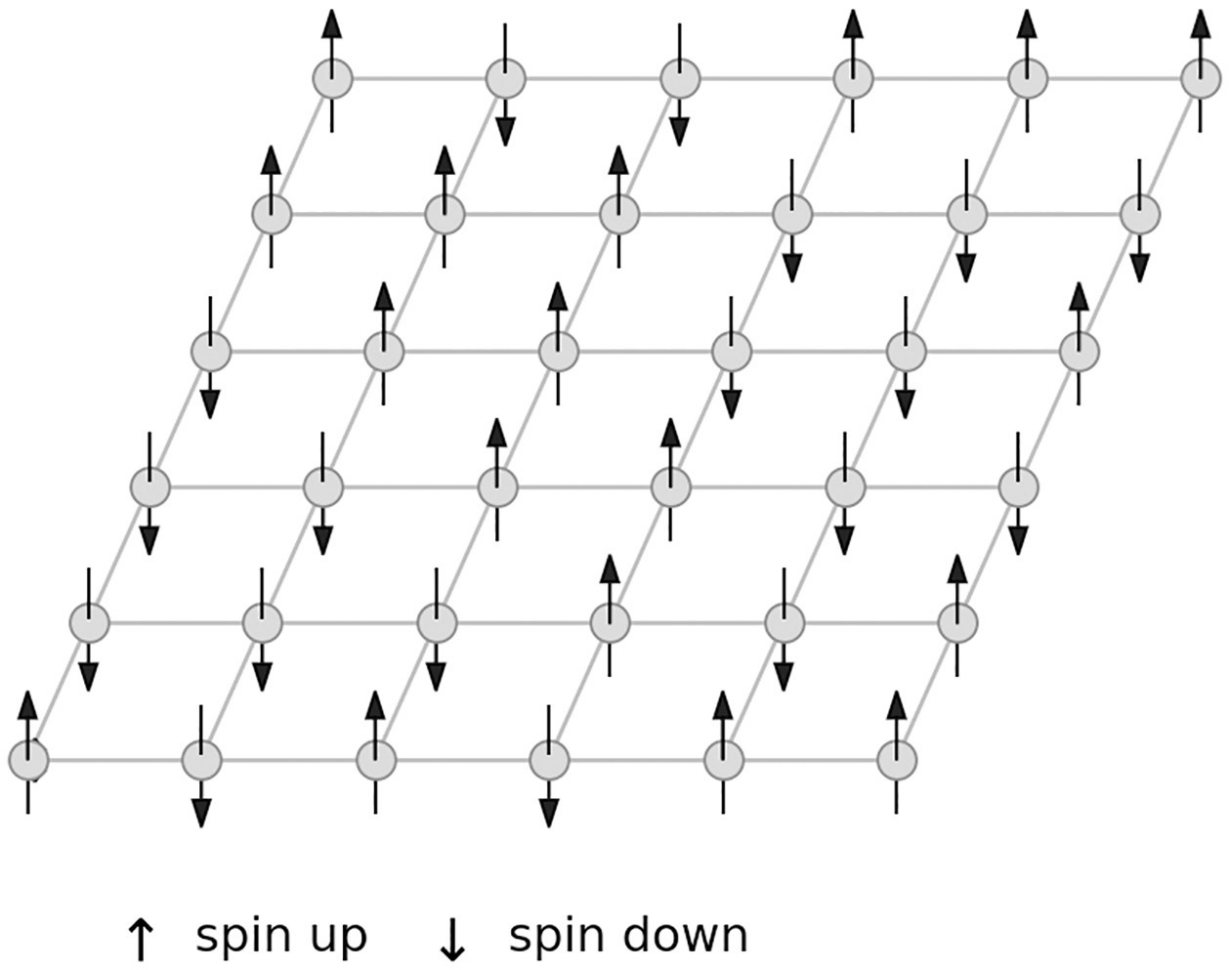
Ising spin field. Illustration of a spin field in the 2D Ising model. An upward arrow marks a node with an upward spin, i.e. *s* = + 1, and a downward arrow marks a node with a downward spin, i.e. *s* = −1. Nodes in the spin field seek spin alignment with their immediate neighborhood. The illustration depicts a regular 2D grid network structure. In our simulations, however, we use random degree mixed networks such as shown in Fig 1 as the underlying network structure.

The Ising model can be described as a system consisting of a collection of spins connected in a grid like structure (see Fig 2). The connections allow spins to interact with their neighboring spins. We refer to the specifics of this neighborhood interaction as the coupling mechanism. Spins can be in two distinct states of which one is spin up and the other is spin down. Seeking a low energy state, spins may flip from one state to another, depending on the potential gain in energy. The magnitude of this potential gain is dependent on the spin’s own state and the states of its neighborhood spins. In mathematical form, the system may be represented by its mean field equation

$$\begin{array}{c} M=\text{tanh}\left[\frac{1}{T}\left(M+H\right)\right] \end{array}$$ (4)

where *M* is the average spin or magnetization, *T* the temperature, and *H* the external magnetic field of the spin system. Note that the above equation is the analytic solution to the 2D Ising model. For our purposes, however, we use a computational version of the Ising model that can account for arbitrary network structures, where we randomly iterate over all spins in the system and derive a probability of flipping a spin by taking the exponential of the negative ratio between the energy *E* that a spin can gain by flipping and the temperature *T* [18, 25]. The potential gain in energy of a spin *s* is given by

$$\begin{array}{c} E=2s(G+H) \end{array}$$ (5)

where *s* is the spin of a particular node in the network and *G* is the sum of all spins in the direct neighborhood of that node. The spin of a node *s* will flip

$$\begin{array}{c} \text{if}\;E\leq 0\;\text{or}\;p<\text{exp}\left(-\frac{E}{T}\right) \end{array}$$ (6)

where *p* is random real number between 0 and 1. Both in its mathematical and computational form, the system exhibits a rich set of bifurcations, notably a pitchfork bifurcation with respect to changes in the temperature *T* and a bifurcation showing the phenomenon of hysteresis with respect to changes in the external magnetic field *H* [10]. Note that this bifurcation may be interpreted as the combination of two saddle-node bifurcations in which equilibrium points appear and disappear in such a way that the system may transition abruptly between two states [14]. While both bifurcations are interesting in their own right, the bifurcation showing the phenomenon of hysteresis is of particular interest for our purpose of studying critical transitions. This type of bifurcation can be used to induce a first order phase transition which shares many features typically associated with critical transitions, namely an abrupt discontinuous jump in the state of system and the phenomenon of hysteresis [26].

**Fig 3:**
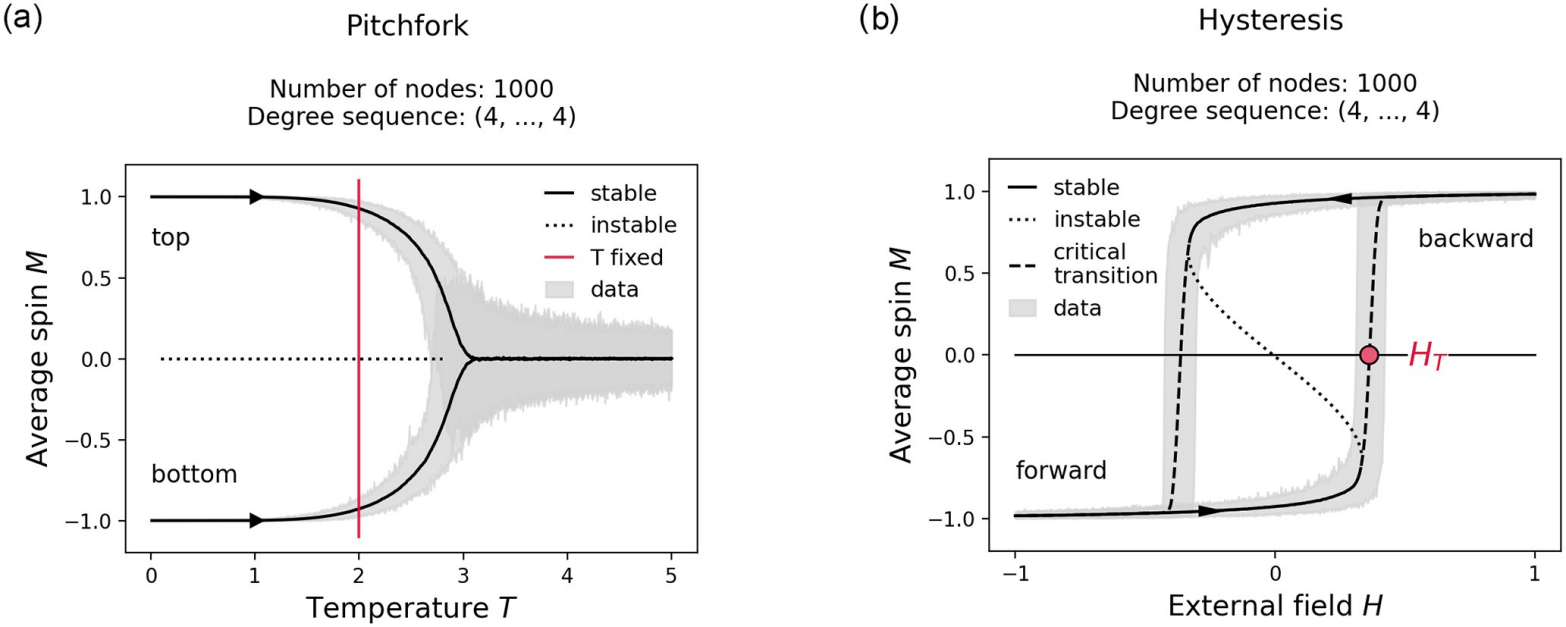
Networked Ising model. Shows two types of bifurcations observed in the networked Ising model. Simulations were performed on random networks of 1000 nodes with homogeneous degree sequence (4, …, 4). The solid and dashed lines indicate the mean transition curve of 1000 runs for which a critical parameter was iteratively increased or decreased to induce the transition. Solid lines mark stable states, dotted lines mark unstable states, and dashed lines mark the critical transition on which we define the tipping point *H*_*T*_. The figure on the left depicts a pitchfork bifurcation with respect to changes in the critical parameter *T* and the figure on the right depicts a bifurcation showing the phenomenon of hysteresis with respect to changes in the critical parameter *H* where the temperature *T* is fixed at 2. Note that this temperature value was selected for illustrative purposes only. In all subsequent simulations, the temperature is set to 1 to ensure a bistable stability landscape for a larger range of degrees. Additional information on how the two bifurcation diagrams relate to each other, for example, in the form of a cusp diagram, can be obtained from [27, 28].

Through Monte Carlo experiments of our computational version of the Ising model, we create transitions curves for differently networked systems. More specifically, for any given network structure we sample multiple transition curves and then regard the mean transition curve as approximation for the true transition curve. Following this methodology, example transitions for both the pitchfork bifurcation and the bifurcation showing hysteresis are presented in Fig 3.

### Simulation setup

To analyze how mixing of different degrees in a network’s degree sequence impacts the transition behavior of a networked system, we devise the following simulation setup: We generate degree sequences with length *N* equal to 1000 through substitution between *m* equal to 2 and 3 distinct degrees. The degree sequences generated by this substitution process are then used to construct random networks following our implementation of the configuration model. For *m* = 2 we generate 1000 transition curves, i.e. one transition curve for every degree substitution. For *m* = 3 we generate a little over 23000 transition curves. Critical transition curves are generated by running our networked implementation of the Ising model on each network. More specifically, we construct transition curves corresponding to the hysteresis bifurcation by fixing the temperature *T* of the system such that the system is locked in a stability landscape with two stable states and then vary the magnitude of an external magnetic field *H* to induce the transition. For all simulations, the temperature *T* is kept constant at 1 while the external magnetic field *H* is iteratively increased in 1000 steps over the closed interval [-10, 10]. From the transition curves, we determine the approximate tipping point *H*_*T*_ by finding the value of the control parameter *H* for which the average spin of the system has passed half of the whole state transition, in our case, where the average spin is greater than 0. The approximate tipping point *H*_*T*_ of the system is then put in relation to the average degree of the degree mixed network.

## Results

**Fig 4:**
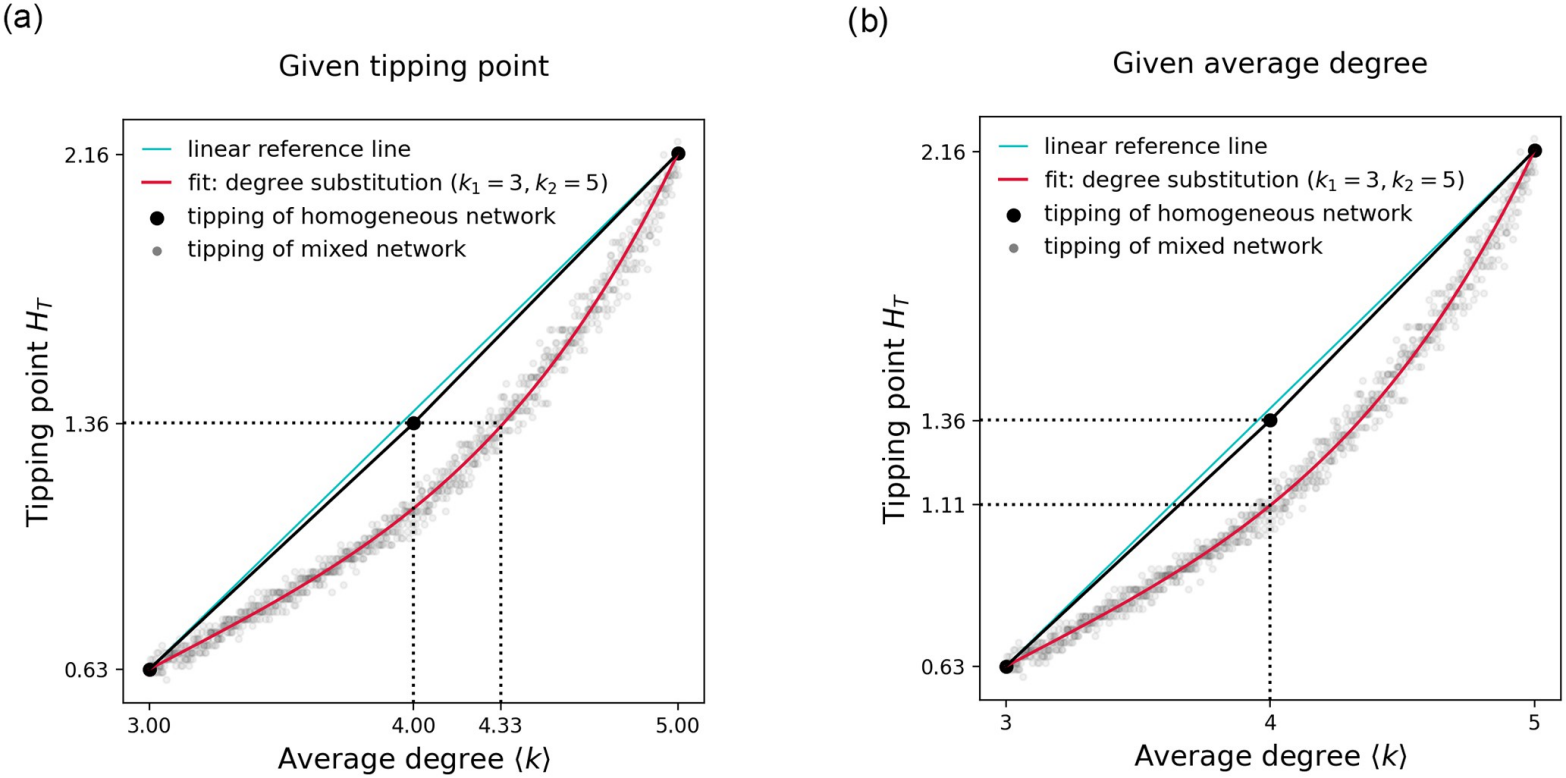
Critical transitions in degree mixed networks. Shows tipping points in networks of 1000 nodes for all possible degree sequence substitution between nodes of degree 3 and 5. The reference line shows that the tipping points of degree homogeneous networks do not follow a perfect linear relationship.

Fig 4 shows how the onset of a critical transition is affected by the degree sequence of a networked system. It compares tipping in networks with degree sequences generated by substituting between two distinct degrees (*k*_1_ = 3, *k*_2_ = 5) with tipping in networks of homogeneous node degree. We find that tipping in degree mixed networks is significantly skewed downwards meaning that critical transitions in mixed networks occur in general sooner than in their degree homogeneous counterparts. In particular, we make the following two observations: Given a fixed tipping point *H*_*T*_ (see Fig 4, left), a mixed network must exhibit a higher average degree compared to an equivalent homogeneous degree network. In the example below, tipping occurs at the control parameter value *H* equal to 1.36 in the degree mixed network with average degree 4.33 compared to a homogeneous degree network with average degree 4. Similarly, given an average degree 〈*k*〉 (see Fig 4, right), a degree mixed network will tip sooner compared to a homogeneous degree network. In the example below, the degree mixed network of average degree 4 tips at the control parameter value *H* equal to 1.11 while the homogeneous degree network of average degree 4 tips at the control parameter value *H* equal to 1.36.

As a side note, this relationship between tipping in mixed and homogeneous degree networks allows us to make inferences about homogeneous degree networks with fractional degrees. For example, given an average degree of 4.33, we know that the mixed system tips at the control parameter value *H* equal to 1.36. If we were able to construct a homogeneous degree network where every node had a fractional degree of 4.33 we can infer that such a system would tip much later, i.e. at a control parameter value *H* greater than 1.36.

**Fig 5:**
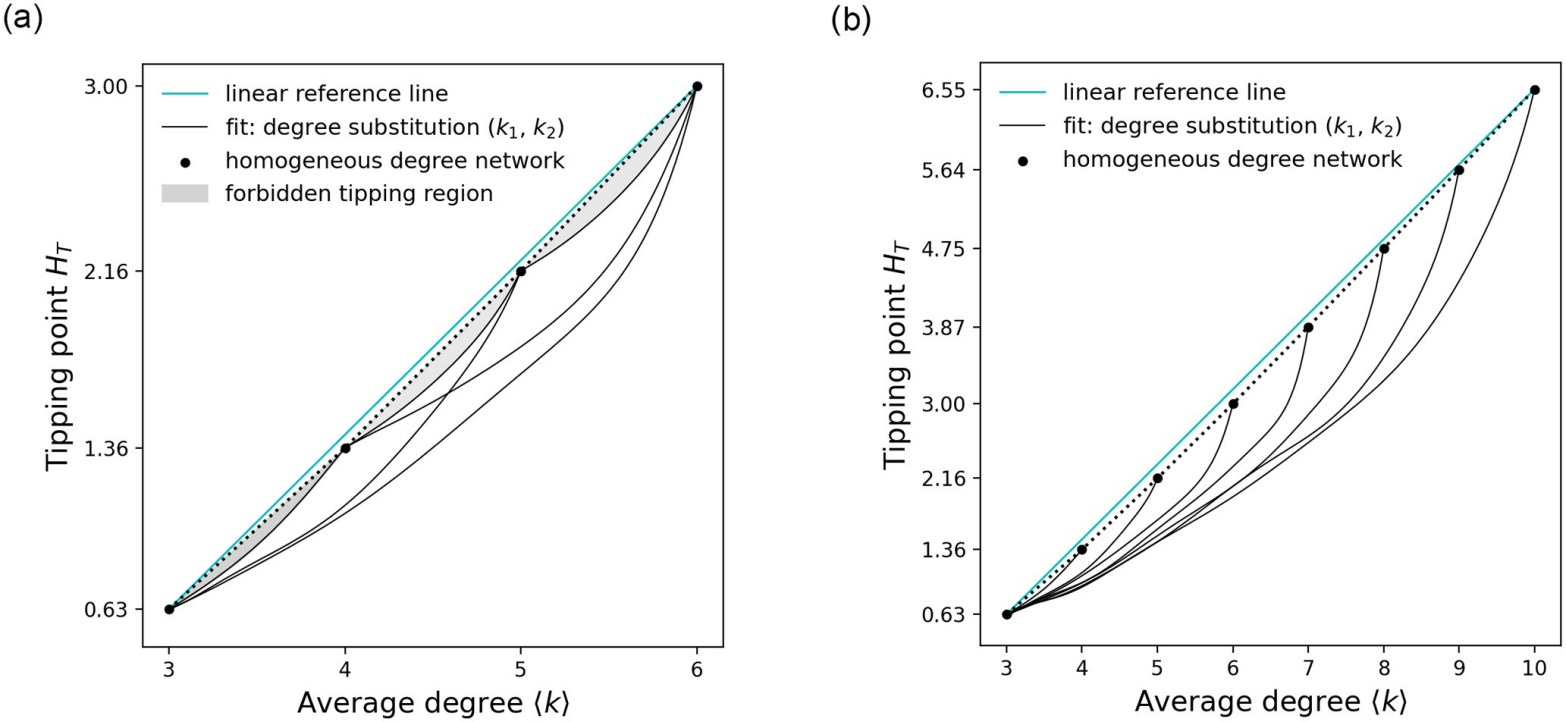
Critical tipping pattern. Shows tipping boundaries created by fitted curves for degree mixed networks between two degrees (*k*_1_, *k*_2_). The gray area marks forbidden tipping regions and the reference line shows that the tipping points of degree homogeneous networks do not follow a perfect linear relationship.

Continuing, we look at how critical transitions behave for various combinations of degree substitutions and find a pattern that seems to persist. Fig 5 shows that the fitted curves representing the approximate tipping points in degree mixed networks relate to each other in a very specific way. The curve corresponding to a degree sequence substitution between two degrees *k*_*i*_ < *k*_*j*_ spans over all curves corresponding to degree sequence substitutions between *k*_*n*_ < *k*_*m*_ where *k*_*i*_ ≤ *k*_*n*_, *k*_*m*_ ≤ *k*_*j*_. These curves mark clear tipping boundaries for a given degree mixed network and allow the identification of possible tipping intersections and forbidden tipping regions when comparing networks with different degree sequences. To illustrate, the fitted curve for the degree mixed networks containing only the degrees (3, 6) spans over both the fitted curve for the degree mixed networks containing degrees (3, 5) and the fitted curve for the degree mixed networks containing degrees (4, 6), which in turn span over the fitted curves (3, 4), (4, 5) and (4, 5), (5, 6) respectively.

This information can be used to narrow down the expected tipping point of a networked system when given the network’s degree sequence. Fig 5 shows that some tipping areas have overlap meaning that they can be reached by multiple different degree combinations, while others may only be reached by one specific degree combination. Note that some tipping areas cannot be reached at all by a degree mixed network as is the case with areas spanned by the curves between two successive integer degrees and the direct connection between them.

**Fig 6:**
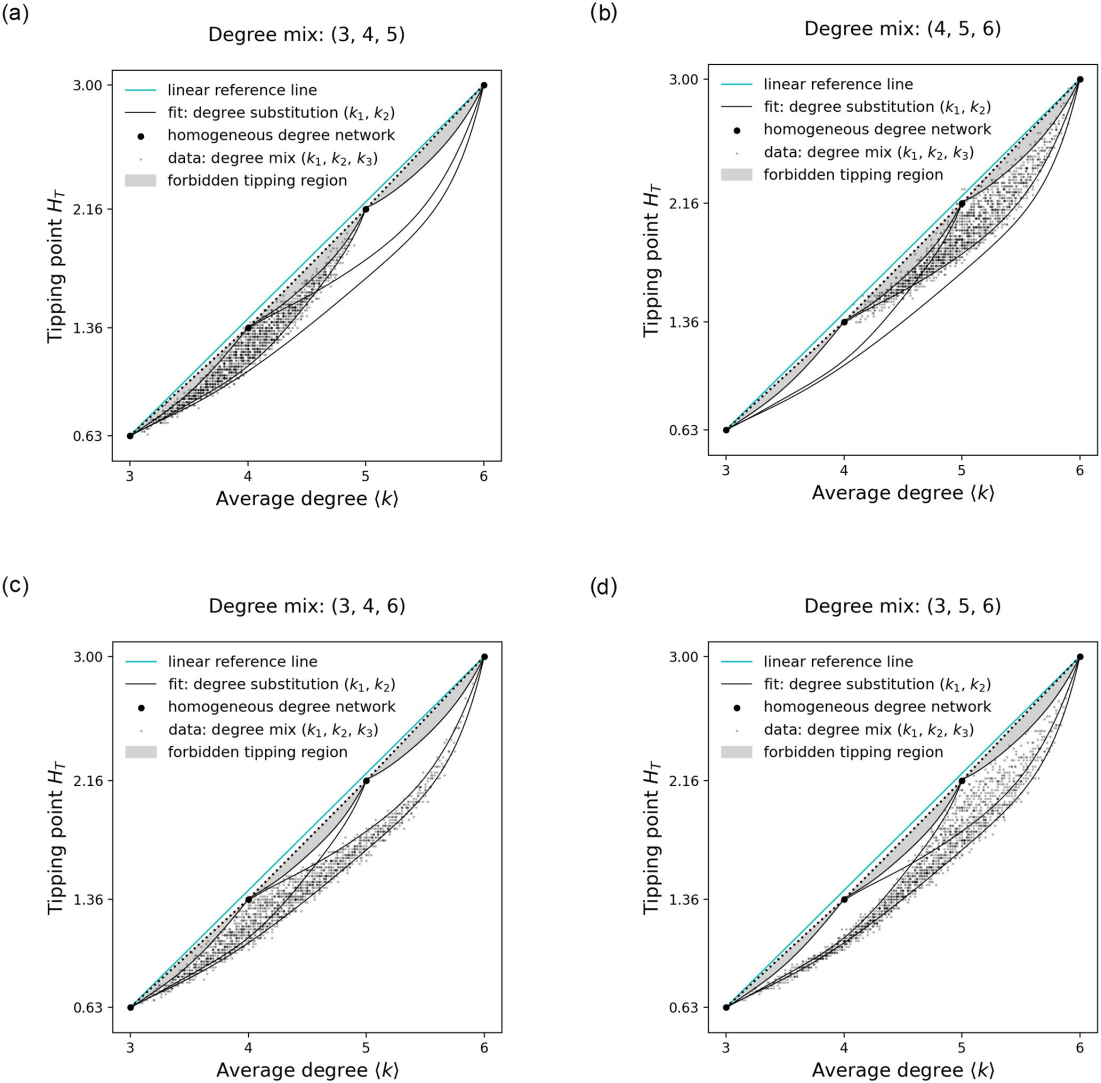
Tipping points in mixed networks containing multiple degrees. Figures show the statistical spread of tipping points *H*_*T*_ generated for degree mixed networks with degree sequences containing three distinct degrees (*k*_1_, *k*_2_, *k*_3_).

We further verified this observation by sampling tipping points over all possible degree sequence combinations between the degree types (3, 4, 5, 6). As can be seen in Fig 6, degree sequences containing different combinations of the degrees (3, 4, 5) all lie in the area spanned by the curves (3, 4), (4, 5) and (3, 5). The tipping point of a combination of the degrees (3, 4, 5) is statistically unlikely to land outside of the area spanned. The same applies to all combinations of three from the degree types (3, 4, 5, 6).

## Discussion

In this paper we analyzed how mixing of different degrees in a network’s degree sequence impacts the transition behavior of a networked system. Our results show that critical transitions in degree mixed networks occur in general sooner than in their degree homogeneous counterparts. More specifically, when presented with two networks of equal average degree where one is composed of a mix of degrees and the other is of homogeneous degree, then the mixed network tips sooner. This suggests that the average degree of network might be a misleading indicator when it comes to estimating the onset of critical transitions in a networked system. The presence of low degree nodes seems to disproportionately reduce a system’s resilience. This could, perhaps, make intuitive sense when looking at how critical transitions are generated in the Ising model. Conceptually speaking, each node in the system experiences two opposing forces. A directive force that tries to push the system into an alternative state and a conservative coupling force that tries to keep the system in its current state. The lower the node degree, the fewer neighbors there are to exert a conservative force on it, causing the node to flip earlier compared to higher degree nodes. Given a sufficiently strong directive force, well distributed low degree nodes can therefore undermine the conservative strength or resilience of the whole networked system since the neighborhood of a higher degree node must essentially compensate for a guaranteed flip of a low degree node in their neighborhood. We further tested the behavior of critical transitions in networks configured with a range of different degree substitutions between two degrees. In this regard, we found that the curves describing the relationships between tipping points and average degree of a mixed network relate in very specific way to each other. A curve corresponding to a degree sequence substitution between two degrees *k*_*i*_ < *k*_*j*_ spans over all curves corresponding to degree sequence substitutions between *k*_*n*_ < *k*_*m*_ where *k*_*i*_ ≤ *k*_*n*_, *k*_*m*_ ≤ *k*_*j*_. This allows us to define, given a network’s degree sequence, tipping areas at which the system is or is not likely to undergo a critical transition. In addition, it allows us to compare different mixed networks in terms of their resilience and make estimates as to where the tipping point would shift if we were to make changes in the network’s degree mix.

As for limitations we want to again emphasize to what kind of systems our results are applicable. The critical transitions generated in this study are induced by a qualitative change in a very specific stability landscape, namely that of hysteresis bifurcation (see Fig 3, right). The critical transitions along this bifurcation are generated by simulations of the Ising model. Conceptually, the Ising model has been used to study many systems outside of its original scientific interest due to it operating on a very general coupling mechanism. Our results are applicable to networked systems that share this mechanism. To specify, the system in question must exhibit the following mechanism: The future state of a node must be dependent on its current state and the current states of its neighbors, to form a conservative force that tries to keep the system in its current state. And the state of a node must be sensitive to a directive force that allows the system to be driven into an alternative state. To give just one example, in a social context this has been related to social versus factual influence, where individuals in an opinion formation process also consider their neighbor’s opinion (social influence or conservative force) when presented with an external information (factual influence or directive force) [18].

In conclusion, the obtained results allow us to make good estimates about a system’s transition behavior when given information about the underlying network structure in terms of its degree sequence. We also see great opportunity for further research regarding other types of bifurcations and network properties relevant to the study of critical transitions and systems resilience.

## Appendix

**Fig 7:**
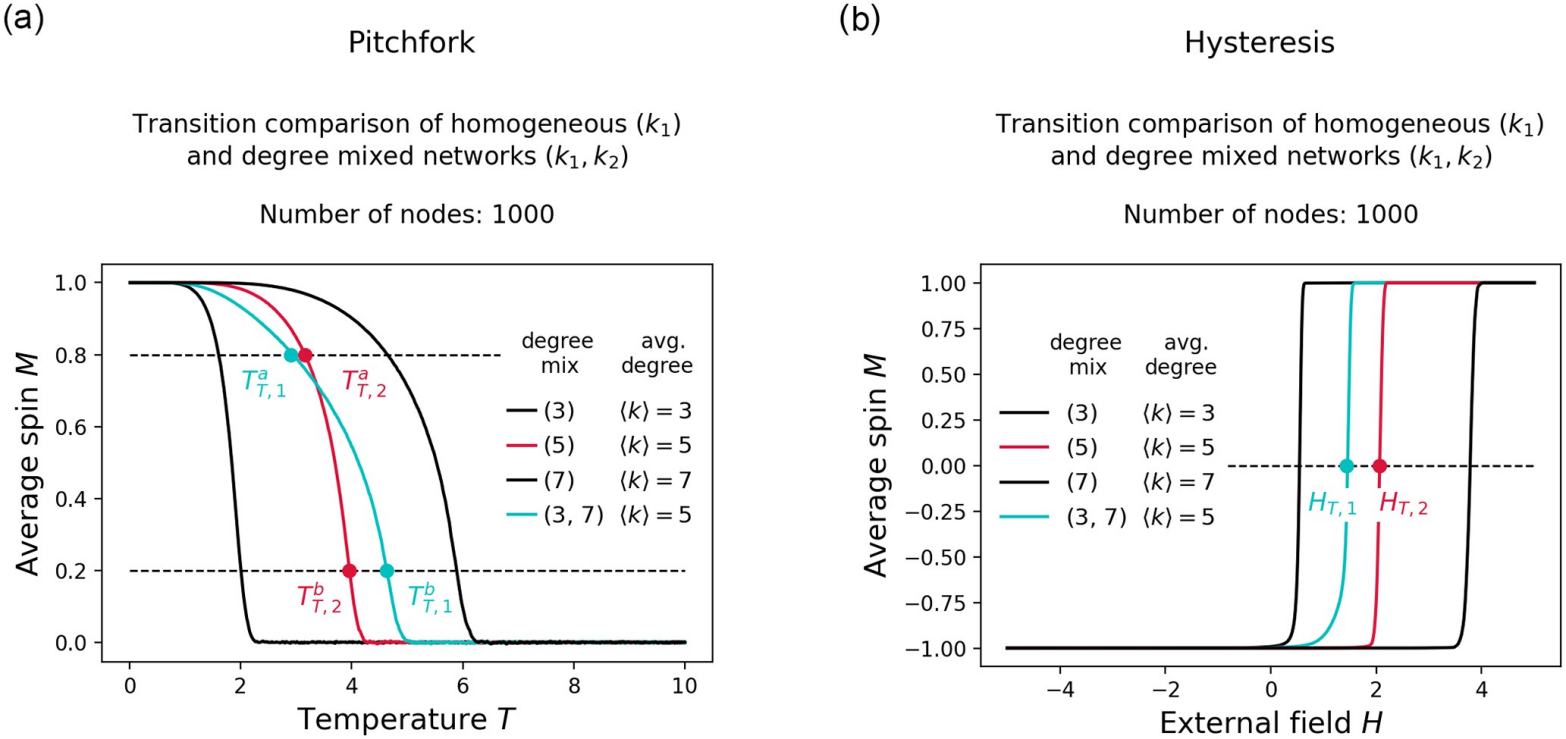
Transition comparison for different bifurcation types in the networked Ising model. Figure on the left shows a smooth state transition along a pitchfork bifurcation where the system is initialized in the top left and the critical parameter *T* is iteratively increased from 0.01 to 10 to induce the transition. Figure on the right shows an abrupt state transition (critical transition) along a bifurcation with hysteresis where the system is initialized on the bottom left and the critical parameter *H* is iteratively increased from -5 to 5 to induce the transition. Tipping point candidates in the pitchfork system are indicated by *T*_*T*_ and by *H*_*T*_ in the hysteresis system. The red line corresponds to a transition on a homogeneous degree network with average degree 5, and the cyan line corresponds to a transition on a degree mixed network consisting of degrees 3 and 7, also with average degree 5.

In the appendix we discuss what to expect for the transition behavior along the other type of bifurcation mentioned in the paper, namely the pitchfork bifurcation. Fig 7 shows a comparison of transition curves for the pitchfork and the hysteresis bifurcation in the networked Ising model. Note that the transition along the pitchfork bifurcation is qualitatively different from the transition along the hysteresis bifurcation. We observe a smooth transition along a stable state in the pitchfork case compared to an abrupt transition (critical transition) between two different stable states in the hysteresis case. This makes it difficult to define a tipping point in the pitchfork case. The system never left its stable state and did thus not tip to an alternative stable state. Additionally, depending on where we draw a reference line for determining tipping points (dotted horizontal lines marking the tipping points $T_{T}^{a}$ and $T_{T}^{b}$ in Fig 7) we may draw different conclusions about the tipping order of a homogeneous network and a degree mixed network with equal average degree (red and cyan lines in Fig 7). We do not face such a problem with the critical transition in the hysteresis case.

In general, we find that both transitions are affected by the degree mix of the underlying network and that the transition starts in general sooner in a degree mixed network than in a homogeneous degree network with equal average degree. However, only in the hysteresis case may we draw a conclusion about the tipping of the system.
