# Correction: Critical transitions in degree mixed networks: A discovery of forbidden tipping regions in networked spin systems

**DOI:** 10.1371/journal.pone.0294198

**Published:** 2023-11-03

**Authors:** Daniel Reisinger, Raven Adam, Marie Lisa Kogler, Manfred Füllsack, Georg Jäger

There are errors in the Funding section. The correct Funding statement is: The authors acknowledge the financial support by the University of Graz.
